# Associations Between Chronotype, Genetic Susceptibility and Risk of Colorectal Cancer in UK Biobank

**DOI:** 10.1007/s44197-025-00399-6

**Published:** 2025-04-10

**Authors:** Huajie Xie, Zhihui Xi, Suqi Wen, Runbei Zhang, Yongfeng Liu, Jiabin Zheng, Huolun Feng, Deqing Wu, Yong Li

**Affiliations:** 1 Guangdong Medical University, Zhanjiang, 524000 China; 2 Department of Gastrointestinal Surgery, Department of Genral Surgery, Guangdong Provincial People’s Hospital (Guangdong Academy of Medical Sciences), Southern Medical University, Guangzhou, 510080 China; 3 Ganzhou Hospital of Guangdong Provincial People’s Hospital, Ganzhou Municipal Hospital, Ganzhou, 341000 China; 4 School of Medicine, South China University of Technology, Guangzhou, 510006 Guangdong China

**Keywords:** Chronotype, Circadian rhythm, Genetic susceptibility, Colorectal cancer, UK Biobank

## Abstract

**Background:**

Sleep problems are common in the general population, with evidence suggesting a link between circadian rhythm disruptions and various health outcomes. However, the role of chronotype in influencing colorectal cancer (CRC) risk, particularly in conjunction with genetic predisposition, remains unclear and warrants further investigation.

**Methods:**

We analyzed data from 295,729 UK Biobank participants, among whom 4305 developed colorectal cancer. Chronotype was self-reported as morning or evening type, and a polygenic risk score for chronotype was generated from 316 genome-wide significant SNPs using 23andMe effect sizes to reduce overlap bias. Colorectal cancer risk was estimated using Cox proportional hazards models adjusted for age, sex, smoking, alcohol consumption, and the Townsend index.

**Results:**

Late chronotype and high polygenic risk were independently associated with an increased risk of CRC. Compared to participants with an early chronotype, those with a late chronotype exhibited a 6.5% increased risk of CRC [HR 1.065, *P* = 0.046]. Similarly, individuals in the high genetic risk group had a 11.0% increased risk compared with those in the low genetic risk group [HR, 1.110, *P* = 0.032]. Stratified analyses revealed that individuals with an intermediate genetic risk who had a late chronotype showed a 17.6% higher risk of CRC [OR, 1.176, *P* = 0.004], whereas those with a high genetic risk had a 25.3% increase [OR, 1.253, *P* = 0.001]. Through analyzing the combined effects of chronotype and PRS, we found that among individuals with an early chronotype, those with intermediate PRS had a 15.4% increased risk of CRC [HR, 1.154, *P* = 0.005], and those with high PRS had a 14.7% increased risk [HR, 1.147, *P* = 0.027].

**Conclusions:**

Our findings highlight the importance of considering circadian rhythm patterns and genetic predispositions when assessing CRC risk, suggesting that chronotype may be associated with CRC risk, but further studies are needed to integrate objective circadian measurements.

**Supplementary Information:**

The online version contains supplementary material available at 10.1007/s44197-025-00399-6.

## Introduction

Colorectal cancer (CRC) ranks is the third most frequently diagnosed cancer with over 1.92 million newly diagnosed cases and 0.9 million deaths annually worldwide [1]. Moreover, this upward trend shows no signs of abating [1, 2]. In recent year, the association between modifiable lifestyle factors, including physical activity, diet patterns, smoking, drinking, obesity, and CRC has attracted increasing attentions [3–7]. Despite significant advances in understanding the risk factors for CRC, including lifestyle behaviors, dietary patterns, and genetic predispositions, recent studies have also highlighted the long-term influence of circadian rhythm on CRC, suggesting that its impact may be as significant as these traditional lifestyle behaviors [8–10].Some evidence has demonstrated that sleep problems are prevalent in the general population. A recent meta-analysis involving over 1.1 million individuals from the United States, Netherlands, and United Kingdom indicated that 13.3% of adult participants reported poor sleep quality [11]. Furthermore, sleep problems are prevalent among both children and the elderly, with rates reaching up to 30% [12, 13].

The patterns of activity/sleep and eating/fasting, are controlled by a circadian clock located in the suprachiasmatic nucleus of the hypothalamus [14]. This biological clock not only regulates daily behavior but also profoundly influences metabolic processes. Disruptions in circadian rhythms, such as those caused by shift work, jet lag, and irregular lifestyle habits, can lead to metabolic disorders and increase the risk of various diseases including colorectal cancer [15]. Previous studies have been limited because most have primarily focused on examining individual sleep characteristics (e.g., sleep duration, insomnia, and daytime napping) and their associations with specific diseases, such as diabetes and hormone-dependent cancers, while overlooking their effects on digestive system cancers [16]. Emerging evidence suggests that long-term circadian disruption, particularly from extended periods of rotating night shift work and exposure to the light at night, is associated with an increased risk of CRC [17, 18]. Furthermore, current research has shown that genetic predisposition may interact with lifestyle behaviors in the development of health outcomes. However, the influence of a healthy sleep pattern, which includes various sleep traits, on the effects of genetic risk for digestive diseases has yet to be thoroughly examined. Although previous studies have investigated the relationship between circadian disruption and colorectal cancer, most have focused on individual behavioral or environmental factors (e.g., shift work, or irregular lifestyles) without simultaneously incorporating self‐reported chronotype and genetic risk. In contrast, our study uniquely combines self-reported chronotype data with a polygenic risk score derived from multiple SNPs to provide a more comprehensive assessment of colorectal cancer risk. This integrated approach enables us to evaluate not only the impact of behavioral factors on cancer risk but also the contribution of genetic predisposition, thereby offering a broader perspective on colorectal cancer etiology.

Therefore, we used a large prospective cohort from the UK Biobank (UKB) dataset. We aimed to explore the association between the chronotype and the risk of CRC. In addition, we applied the SNPs used in a previous study to construct chronotype and assess their genetic susceptibility. Our study sample consisted of 295, 729 individuals and we considered a comprehensive range of CRC risk factors, including traditional lifestyle factors, sleep patterns, family disease history, and other related covariates.

## Research Design and Methods

### Study Population

**Fig. 1:**
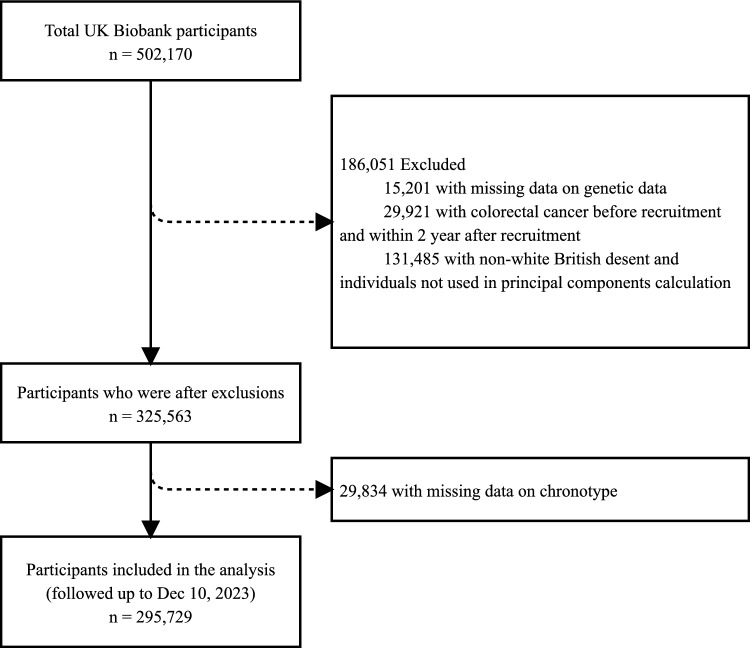
Flowchart for the selection of the analyze d study sample from the UK Biobank study

Data were obtained from the UKB, a population-based cohort of more than 500, 000 adults in the UK between the ages of 40 and 70 years at recruitment who were followed up prospectively [19]. Individuals from the UKB were recruited between 2006 and 2010 and followed up prospectively with linkage to health data records through April 2024. At the time of the baseline study visit, participants used a touch screen to provide detailed information about chronotype, smoking history and alcohol intake among other lifestyle factors. Whole-exome sequencing of blood DNA was performed among a subset of individuals. Genetic associations with colorectal cancers were obtained from a UKB study [20]. The UKB research was approved by the North West Multicenter Research Ethical Committee. All participants provided written informed consent to participate in the study. In the present study, we excluded participants with missing values in the genotyping data (n = 15, 201), those diagnosed with bowel cancer before recruitment, those diagnosed with colorectal cancer or deceased within two years after recruitment (n = 29, 921), and those with missing data on the primary exposure factor chronotype (n = 29, 834) at baseline. The excluded individuals were not White British (ID 21000) and individuals not used in the principal components calculation (ID 22020) from our analysis (n = 131, 485), as GWASs have primarily focused on white British populations, leaving 295, 729 participants for the primary analysis (Fig. 1).

### Definition of Genetic Predisposition

**Fig. 2:**
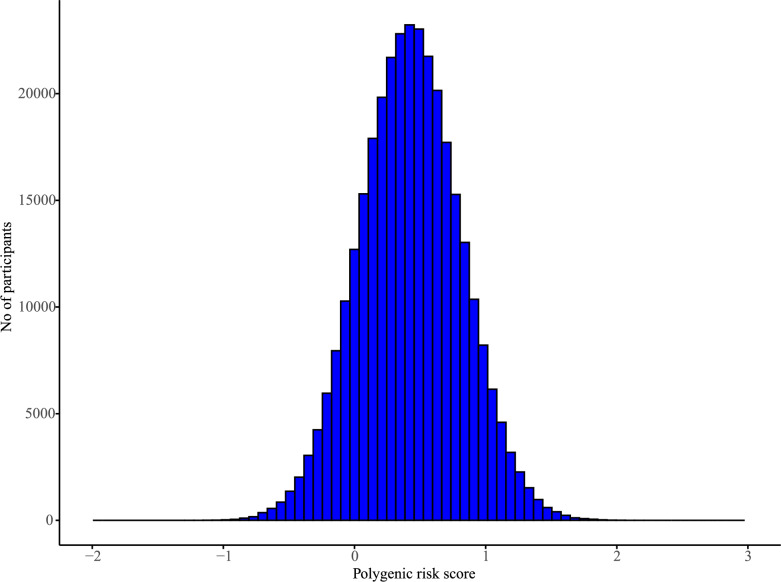
Distribution of Polygenic Risk Scores Based on 316 Chronotype-Related SNP

To estimate genetic predisposition, PRS for colorectal cancer in each participant were constructed. First, We identified 316 SNPs associated with chronotype from the study by Yuan et al. (PMID: 36,093,575), which were reported in their supplementary materials and had reached genome-wide significance (P < 5 × 10⁻⁸) [9, 21]. These SNPs were then retrieved from the UK Biobank (UKB) genetic dataset, and a polygenic risk score (PRS) was calculated to assess genetic susceptibility to chronotype-related colorectal cancer risk. All genetic data used in this study were obtained from the UK Biobank. Detailed information on the selected SNPs is provided in Table S1. To process the genetic data, we used the UK Biobank Research Analysis Platform (UKB-RAP) for SNP extraction and PRS computation. First, we utilized the bgenix command to extract the target SNPs from the UKB bgen file subset. The extracted genotype data were then converted into a standard tabular format using plink2, facilitating downstream analysis. In Table S1, we present the extracted SNPs and their relevant parameters, including Beta_meta, SE_meta, Beta_23, and SE_23. To ensure consistency in PRS calculation, we followed previous studies and selected Beta_23 as the effect size, as it was derived from the 23andMe dataset and reflects the SNP’s impact on chronotype. Each SNPs was recoded as 0, 1, or 2 according to the number of risk alleles. These SNPs were then weighted by the regression coefficients obtained from the Beta_23 data to calculate the PRS:

$$\text{PRS }= \left({\upbeta }_{1} \times {\text{ SNP}}_{1} + {\upbeta }_{2} \times {\text{ SNP}}_{2} + \cdots + {\upbeta }_{\text{n}} \times {\text{ SNP}}_{\text{n}}\right)$$

n = No. of SNPs, as shown in Fig. 2. Furthermore, we classified individuals into three groups as follows: high (quintile 5), intermediate (quintile 2–4), or low (quintile 1) PRS, as previously described [22, 23].

### Chronotype

The chronotype (ID 1180) was asked, ‘Do you consider yourself to be?’ with response options of ‘Definitely a “morning” person’, ‘More a “morning” than an “evening” person’, ‘Definitely an “evening” person’, ‘Do not know’ and ‘Prefer not to answer’. ‘Do not know’ or ‘Prefer not to answer’ responses were treated as missing values. We encoded ‘Definitely a “morning” person’ and ‘More a “morning” than an “evening” person’ as early chronotype, and ‘More an “evening” than a “morning” person’ and ‘Definitely an “evening” person’ as late chronotype. Specifically, participants who selected “Definitely a morning person” or “More a morning than an evening person” were defined as having an early chronotype, whereas those who selected “More an evening than a morning person” or “Definitely an evening person” were defined as having a late chronotype.

### Confounders

We considered several factors as potential confounders of the association between chronotype, PRS, and colorectal cancer risk: age (ID 21022), sex (ID 31), body mass index (BMI) (ID 21001), smoking (ID 20116), alcohol intake (ID 1558) and Townsend index (ID 22189). Table S1 provides detailed information on the confounders. BMI was derived from the weight and height measured when the participants attended the initial assessment center. The Townsend deprivation index, calculated immediately prior to participation in the UKB, assigns each participant a score corresponding to the output area in which their postcode is located. Detailed information about confounders is shown in Table S2.

### Outcome

The primary outcome, as shown in Table S3, was the presence of all colorectal cancers. Incident colorectal cancer and death were defined in the UK Biobank by means of enrollment interview and linking participant information to national electronic health and death records. Participants’ electronic health records (EHRs), including inpatient International Classification of Disease (ICD- 10) diagnosis codes, were integrated into the UKB. Detailed coding information related to colorectal cancer is provided in Table S2. Individuals were defined as having colorectal cancer based on at least one of the following criteria: (1) Hospitalization for an ICD- 10 code for malignant neoplasm of the colon (C18, C18.0–18.7, C18.9, C19); (2) Hospitalization for an ICD- 10 code for malignant neoplasm of the rectum (C20); (3) Hospitalization for an ICD- 10 code for malignant neoplasm of the anus and anal canal (C21, C21.0–21.2, C21.8). Death was ascertained via linkage to death registries. We computed the follow-up time from the date of attendance to the first diagnosis date, date of death, or last registered follow-up (12/10/2023), whichever occurred first.

### Statistics

We used the chi-square test to compare categorical features, the t-test for normally distributed continuous variables, and the Mann–Whitney U test for non-normally distributed continuous variables. Categorical variables were reported as numbers and percentages, and continuous variables as means (SD) or medians (IQR), depending on their distribution. For comparisons involving more than two groups of normally distributed continuous variables, we used ANOVA. Patients with missing data were excluded, and analyses were conducted only on the complete datasets.

In Cox proportional hazards regression models, we employed a stepwise adjustment approach for covariates. Age and Sex were included in the first adjustment model as these are fundamental and non-modifiable demographic characteristics that are routinely controlled for in epidemiological studies. The second adjustment model further incorporated smoking status and alcohol intake, which are modifiable lifestyle factors with a more direct association with colorectal cancer risk. This stepwise adjustment strategy allows for the differentiation of the effects of demographic factors and lifestyle behaviors on the study outcomes, facilitating an assessment of the incremental impact of lifestyle variables after controlling for fundamental characteristics. Hazard ratios (HR) with 95% confidence intervals (CI) were used to assess the relative risk over the follow-up period, providing insights into the impact of chronotype and genetic predispositions.

Stratified analyses using logistic regression models were conducted to further investigate the interaction between genetic risk and chronotype on CRC risk, with results stratified by chronotype and PRS. This allowed for the estimation of CRC risk within each chronotype group, as well as within each PRS category.

Furthermore, the combined effects of the PRS and chronotype were assessed by creating an interaction term. Joint effects were estimated using Cox models adjusted for age, sex, smoking status, and alcohol intake. The HR and 95% CI were calculated for each level of the combined variable to quantify the relative risk of CRC.

All statistical analyses were performed using R version 4.3.2, with two-sided tests and statistical significance was set at *P* values < 0.05.

## Result

### Baseline Characteristics of the Study Population

Of the 295, 729 participants included in our study, there were 157, 698 females and 128, 587 males. The baseline characteristics of the included participants are shown in Table 1. The median age was 56.69 ± 8.00 years. During a median follow-up of 15.06 ± 1.22 years, there were 4305 cases of incident CRC with an estimated prevalence of CRC was 1.46%. Compared with non-CRC participants, subjects with CRC were characterized by a higher level of smoking history. Compared to participants without CRC, those who developed incident CRC were more likely to be older, male, obesity, excessive alcohol drinkers and smokers. The distribution of chronotype across the genetic risk categories is presented in Table S4.

**Table 1:** Baseline characteristics of the UK Biobank population, separated by CRC status

Characteristics	Total	Non-CRC	CRC	*P* value
	*n* = 295,729	*n* = 291,424	*n* = 4305	
Age, y	56.69 ± 8.00	56.63 ± 8.00	60.38 ± 6.71	< 0.001
Sex, *n* (%)				< 0.001
Female	160,667 (54.3)	158,714 (54.5)	1953 (45.4)	
Male	135,062 (45.7)	132,710 (45.5)	2352 (54.6)	
BMI, kg/m^2^	27.35 ± 4.72	27.34 ± 4.72	27.98 ± 4.78	< 0.001
Smoking status				< 0.001
Never	161,155 (54.5)	159,158 (54.6)	1997 (46.4)	
Previous	105,036 (35.5)	103,168 (35.4)	1868 (43.4)	
Current	29,538 (10.0)	29,098 (10.0)	440 (10.2)	
Alcohol intake frequency, *n* (%)				< 0.001
Daily or almost daily	64,198 (21.7)	63,009 (21.6)	1189 (27.6)	
3–4 times per week	72,427 (24.5)	71,467 (24.5)	960 (22.3)	
1–2 times per week	78,061 (26.4)	77,040 (26.4)	1021 (23.7)	
1–3 times per month	32,589 (11.0)	32,174 (11.0)	415 (9.6)	
Special occasions only	29,975 (10.1)	29,538 (10.1)	437 (10.2)	
Never	18,479 (6.2)	18,196 (6.2)	283 (6.6)	
Townsend index	− 1.64 ± 2.89	− 1.64 ± 2.89	− 1.67 ± 2.89	0.447
Chronotype, *n* (%)				0.814
Early chronotype	186,635 (63.1)	183,926 (63.1)	2709 (62.9)	
Late chronotype	109,094 (36.9)	107,498 (36.9)	1596 (37.1)	
Polygenic risk score				0.065
Low	59,232 (20.0)	58,429 (20.0)	803 (18.7)	
Immediate	177,440 (60.0)	174,826 (60.0)	2614 (60.7)	
High	59,057 (20.0)	58,169 (20.0)	888 (20.6)	

Data are presented as mean ± SD unless otherwise specified. CRC, colorectal cancer

^*^Difference between non-CRC and CRC participants, one-way ANOVA or chi-squared test

### PRS, Chronotype, and CRC Risk

To assess the independent effects of both chronotype and PRS on CRC risk, separate analyses were conducted for each factor. The main effects of PRS and chronotype on the risk of CRC are presented in Table 2. Compared to individuals with an early chronotype, those with a late chronotype had a higher risk of developing colorectal cancer [HR (95% CI), late vs. early chronotype: 1.083 (1.018, 1.153), *P* = 0.011, Model 1]. This association remained significant after adjusting for age, sex, smoking frequency, and alcohol intake [HR (95% CI), late vs. early chronotype: 1.065 (1.001, 1.134), *P* = 0.046, Model 2]. We found that the late chronotype consistently contributed to an elevated risk of colorectal cancer, regardless of the additional covariates controlled for in each model. Moreover, PRS, whether intermediate or high, also showed statistically significant associations with an increased risk of colorectal cancer across all the models. In Model 1, individuals in the high genetic risk category had a significantly higher risk than those in the low genetic risk group [HR (95% CI), high vs. low: 1.112 (1.011, 1.223), *P* = 0.029], and this association persisted after further adjustments in Model 2 [HR (95% CI), high vs. low: 1.110 (1.001, 1.221), *P* = 0.032]. Similarly, individuals with intermediate genetic risk also showed a modestly increased CRC risk compared with the low-risk group in Model 1 [HR (95% CI), intermediate vs. low: 1.089 (1.006, 1.179), *P* = 0.035], and this association remained stable after adjustments in Model 2 [HR (95% CI), intermediate vs. low: 1.088 (1.006, 1.178), *P* = 0.036].

**Table 2:** Hazard ratios and 95% CIs for colorectal cancer, separated by polygenic risk score and chronotype

Characteristics	CRC/non-CRC	Model 1^a^		Model 2^b^	
		HR (95% CI)	*P* value	HR (95% CI)	*P* value
PRS					
Low^c^	803/58,429	1 [Reference]	NA	1 [Reference]	NA
Intermediate	2614/174,826	1.089 (1.006,1.179)	0.035	1.088 (1.006,1.178)	0.036
High	888/58,169	1.112 (1.011,1.223)	0.029	1.110 (1.001,1.221)	0.032
Chronotype					
Early chronotype	2709/183,926	1 [Reference]	NA	1 [Reference]	NA
Late chronotype	1596/107498	1.083 (1.018,1.153)	0.011	1.065 (1.001,1.134)	0.046

*CRC*, colorectal cancer, *PRS*, Polygenic risk score, *HR* Hazard ratio, *NA* Not applicable

^a^Model 1 was adjusted for age (continuous), sex

^b^Model 2 was adjusted for age (continuous), sex, smoking frequency, alcohol intake and Townsend index

^c^Set the Low value of PRS as the reference

### Stratified Analysis of PRS and Chronotype in CRC Risk

No significant differences were observed in the stratified analysis of the genetic risk and chronotype (Table 3). However, in the early chronotype, individuals with immediate or high genetic risk showed a significantly higher risk of colorectal cancer compared to those with an early chronotype [OR (95% CI), intermediate vs. low: 1.157 (1.045, 1.248), *P* = 0.005; OR (95% CI), high vs. low: 1.149 (1.016, 1.262), *P* = 0.027]. This association showed no significant differences between PRS among individuals with a late chronotype in Table 3a.

**Table 3:** Associations between polygenic risk score, chronotype, and colorectal cancer

Chronotype				
PRS	Early chronotype	*P* value	Early chronotype	*P* value
	OR (95% CI)		OR (95% CI)	
(a)				
Low (reference group)	1 [Reference]	NA	1 [Reference]	NA
Immediate	1.157 (1.045, 1.248)	0.005	0.984 (0.856, 1.114)	0.801
High	1.149 (1.016, 1.262)	0.027	1.048 (0.892, 1.203)	0.552

PRS						
Chronotype	Low	*P* value	Intermediate	*P* value	High	*P* value
	OR (95% CI)		OR (95% CI)		OR (95% CI)	
(b)						
Early chronotype (reference group)	1 [Reference]	NA	1 [Reference]	NA	1 [Reference]	NA
Late chronotype	1.193 (1.032,1.32)	0.016	1.030 (0.954,1.105)	0.812	1.086 (0.944,1.22)	0.240

(a) Odds ratios and 95% CIs for the association between Polygenic risk score and colorectal cancer, separated by chronotype. (b) Odds ratios and 95% CIs for the association between chronotype and colorectal cancer, separated by the Polygenic risk score

Logistic regression analysis adjusted for Age, Sex, BMI, Smoking status, Alcohol intake frequency and Townsend index

We further analyzed the association between chronotype and colorectal cancer risk across different polygenic risk categories, as shown in Table 3b. In the low genetic risk category, individuals with late chronotype had a significantly higher risk of colorectal cancer than those with early chronotype [OR (95% CI), late vs. early chronotype: 1.193 (1.032, 1.320), *P* = 0.016]. Although the results did not show statistically significant differences, we observed a trend of increasing colorectal cancer risk as genetic risk increased from intermediate to high. Specifically, in the intermediate genetic risk group, the risk of colorectal cancer in individuals with the late chronotype was not significantly different from that in individuals with the early chronotype [OR (95% CI), 1.030 (0.954, 1.105), *P* = 0.812]. In the high genetic risk group, individuals with a late chronotype showed a slightly increased risk [OR (95% CI), 1.086 (0.944, 1.220), *P* = 0.240], although the difference was not statistically significant.

### Combing Effect of PRS and Chronotype on CRC Risk

**Fig. 3:**
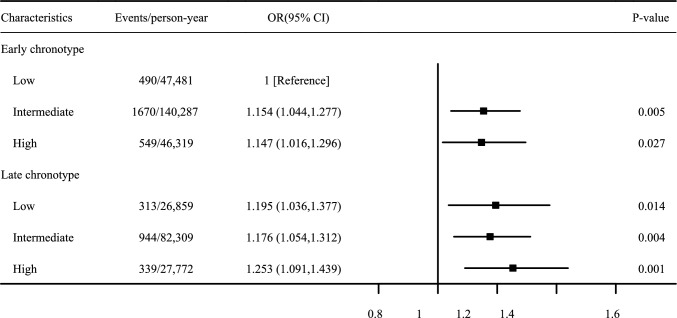
The combined effect of PRS and Chronotype on the risk of incident CRC, adjusted by age, sex, smoking status, alcohol intake frequency and Townsend index

To assess the combined effects of PRS and chronotype on the risk of CRC, we performed analyses the risk across different chronotype and PRS levels, as shown in Fig. 3 and Figure S1. Among individuals with an early chronotype, those with intermediate PRS had a 15.4% increased risk of CRC compared to those with low PRS [HR (95% CI), intermediate vs. low: 1.154 (1.044, 1.277), *P* = 0.005], whereas those with high PRS had a 14.7% higher risk [HR (95% CI), high vs. low: 1.147 (1.016, 1.296), *P* = 0.027]. Similarly, among individuals with a late chronotype, those with intermediate PRS had a 17.6% increased CRC risk compared with those with low PRS [HR (95% CI), intermediate vs. low: 1.176 (1.054, 1.312), *P* = 0.004], and those with high PRS had a 25.3% increased risk [HR (95% CI), high vs. low: 1.253 (1.091, 1.439), *P* = 0.001].

## Discussion

In this large-scale cohort study based on the UKB, we found that both the late chronotype and high polygenic risk were associated with an increased risk of CRC. Both environmental circadian disruptions (e.g., shift work, prolonged light exposure) and mutations in core circadian clock genes have been shown to promote the initiation and progression of colorectal cancer. Studies indicate that shift work or circadian disruption can accelerate APC loss, thereby activating the Wnt/β-catenin signaling pathway and promoting colorectal carcinogenesis [24]; similarly, mutations in genes such as PER2 and CLOCK can lead to aberrant activation of the Wnt pathway [25]. This suggests that both factors may act via the same pathway. Although each factor independently promotes cancer development, when they converge on the same signaling pathway, their combined effect may reach a “saturation” level rather than being simply additive. Our analysis did not reveal a statistically significant interaction between chronotype and polygenic risk, suggesting that the effects of these factors on colorectal cancer risk operate independently rather than synergistically. In other words, regardless of whether individuals have low, intermediate, or high polygenic risk, the impact of chronotype (whether morning or evening type) remains relatively consistent. This independent mode of action may partly explain why the combined hazard ratio in our analysis does not markedly exceed that observed in the PRS-alone analysis, as the risk contributions of the two factors do not simply multiply when combined, which may reflect a saturation effect due to their convergence on the same pathway. Further studies are warranted to validate this hypothesis. Additional analyses within the groups of early and late chronotype carriers showed that individuals with intermediate and high levels had a higher risk of CRC than those who reported low genetic risk.

Dysregulation of circadian clock genes has been increasingly recognized as a factor in cancer development, including CRC. Previous research suggests that alterations in circadian clock function may influence DNA repair mechanisms, metabolic homeostasis, and immune system regulation, all of which are essential for maintaining cellular stability and preventing cancer [26–28]. Our findings observed that both the late chronotype and polygenic risk were independently associated with an increased risk of CRC. Specifically, individuals with late chronotype had a 6.5% higher CRC risk, supporting studies suggesting that differences in chronotype may leads to metabolic dysfunction and heightened cancer risk [9, 10, 29].

Previous research has shown that genetic variants in the CLOCK1 gene significantly increase the risk of CRC [30]. Another study found that the rs37436997 SNP in RORA was significantly associated with an increased risk of CRC occurrence and outcome [31]. However, these studies have mainly focused on the independent effects of genetic variants in core circadian rhythm genes on CRC susceptibility and prognosis. In this study, we constructed a polygenic score based on SNPs related to chronotype to provide more comprehensive and reliable results. Our findings indicate that individuals with both late chronotype and intermediate-to-high genetic risk exhibit significantly elevated CRC risk. Specifically, individuals with late chronotype and intermediate polygenic risk had a 17.6% increased risk of CRC, while those with high polygenic risk exhibited a 25.3% increase. These findings support the hypothesis that circadian clock gene dysregulation may interact with genetic vulnerabilities, thereby increasing CRC risk [32, 33].

Our results show that late chronotype and polygenic risk independently affected CRC risk, with no significant interaction observed between the two factors. To assess whether chronotype was associated with PRS and to evaluate potential multicollinearity, we conducted additional analyses. Linear regression showed no significant correlation between the two variables. Variance inflation factor (VIF) analysis in multivariate Cox and logistic regression models yielded values of 1.016 for chronotype and 1.000 for PRS, well below the threshold for multicollinearity concerns (VIF > 5). These findings confirm that chronotype and PRS independently contribute to CRC risk without mutual interference. Specifically, individuals with late chronotype and low genetic risk had a 19.3% increased CRC risk, suggesting that differences in chronotype may play a role in CRC susceptibility among low genetic risk individuals [31]. In contrast, for those with intermediate and high genetic risk, the late chronotype did not significantly increase CRC risk, suggesting that genetic predisposition is the primary driver of CRC risk in these groups [34].Moreover, we found that late chronotype in low genetic risk individuals was significantly associated with an increased risk of CRC, supporting the hypothesis that late chronotype individuals may exhibit different metabolic and immune function profiles, which could potentially contribute to CRC risk [35]. In contrast, for individuals with intermediate or high genetic risk, genetic predisposition may play a more prominent role in CRC risk, while variations in chronotype may have a relatively smaller impact [36, 37]. We hypothesize that for individuals with a late chronotype or medium-to-high genetic risk, the independent effects of these factors are strong enough to mask any potential synergistic effects. This pattern has also been observed in other cancer studies, where genetic predisposition dominates cancer risk and reduces the influence of behavioral factors such as differences in chronotype [37–39]. However, for individuals with a low genetic risk, chronotype may be associated with CRC risk, potentially through its influence on metabolic and immune pathways. Future studies should explore whether integrating chronotype into CRC prevention strategies may reduce CRC risk in genetically susceptible populations [40]. Our study found that both late chronotype and high polygenic risk score independently increase CRC risk, but previous studies have reported inconsistent findings. Chen et al. [8] found no association between chronotype and CRC using the UK Biobank dataset. This discrepancy may be due to differences in study design. Chen et al. categorized individuals based on a health score without integrating genetic susceptibility, whereas our study combines chronotype with PRS, providing a broader perspective on CRC risk. Additionally, Yuan et al. [9] used Mendelian randomization and reported that morning chronotype was associated with a lower CRC risk. However, their study focused on genetically predicted chronotype, while our study examines both self-reported chronotype and genetic susceptibility. These approaches address different research questions and should be seen as complementary rather than contradictory.

Our study has several strengths. First, it leverages a large-scale prospective cohort from the UKB, allowing for a robust analysis of the joint effects of chronotype and polygenic risk on CRC susceptibility. The large sample size enhance the statistical power and generalizability of our findings to broader populations. Additionally, our analysis accounted for a wide range of confounding factors, including lifestyle behaviors and genetic variations, which strengthened the validity of the results. Second, we adopted a comprehensive approach to assess chronotype using multiple genetic variants, providing a more nuanced understanding of how chronotype interacts with genetic risk in influencing CRC development. This study is among the first to examine the interplay between genetic predisposition and sleep patterns in the context of CRC, contributing to the growing body of research on the role of circadian rhythm in cancer development. However, our study also had some limitations. First, the assessment of chronotype was based on self-reported data, which might have introduced recall bias and misclassification, and may not precisely reflect the participants’ true biological clock. Furthermore, as our study is not a continuously monitored prospective study with frequent follow-ups, the self-reported chronotype might not capture the dynamic changes in circadian rhythms over time. Moreover, our analysis focused on individuals of White British ancestry, limiting the generalizability of our findings to other ethnic groups. Future research should include more diverse populations to validate our results across different genetic backgrounds.

## Conclusion

In conclusion, both late chronotype and high PRS were independently associated with an increased risk of CRC. Individuals with a late chronotype exhibited a higher risk of CRC compared to those with an early chronotype even after adjusting for potential confounders. Similarly, participants in the high PRS group had a significantly elevated CRC risk compared with those in the low PRS group. Our stratified analysis revealed that the combination of late chronotype and high genetic risk further increased the likelihood of CRC development, suggesting a potential cumulative effect. These findings underscore the importance of considering both circadian rhythm patterns and genetic predispositions when assessing CRC risk. Future studies should further investigate the biological mechanisms underlying these associations and explore whether maintaining a healthy circadian rhythm can mitigate the risk of CRC.

## Supplementary Information

Below is the link to the electronic supplementary material.

Supplementary file1 (DOCX 47 KB)

Supplementary file2 (DOCX 14 KB)

Supplementary file3 (DOCX 14 KB)

Supplementary file4 (DOCX 14 KB)

Supplementary file5 (PDF 102 KB)

## Data Availability

The UK Biobank resource is available to researchers upon completion of the registration form in the Access Management System (AMS-https://bbams.ndph.ox.ac.uk/ams/).

## Abbreviations

BMI: Body Mass Index
PRS: Polygenic risk score
CRC: Colorectal cancer
CI: Confidence interval
HR: Hazard ratio
OR: Odds ratio
UKB: UK biobank
