# Correction: Associations Between Chronotype, Genetic Susceptibility and Risk of Colorectal Cancer in UK Biobank

**DOI:** 10.1007/s44197-025-00430-w

**Published:** 2025-06-10

**Authors:** Huajie Xie, Zhihui Xi, Suqi Wen, Runbei Zhang, Yongfeng Liu, Jiabin Zheng, Huolun Feng, Deqing Wu, Yong Li

**Affiliations:** 1 Guangdong Medical University, Zhanjiang, 524000 China; 2 Department of Gastrointestinal Surgery, Department of Genral Surgery, Guangdong Provincial People’s Hospital (Guangdong Academy of Medical Sciences), Southern Medical University, Guangzhou, 510080 China; 3 Ganzhou Hospital of Guangdong Provincial People’s Hospital, Ganzhou Municipal Hospital, Ganzhou, 341000 China; 4 School of Medicine, South China University of Technology, Guangzhou, 510006 Guangdong China

**Correction to: Journal of Epidemiology and Global Health (2025) 15:57**

10.1007/s44197-025-00399-6

When this article was first published, the name of the 4th author was mistakenly given as WenRunbei Zhang, while the correct name is Runbei Zhang.

The Original Article has been corrected.

