# Supplementary material for: Associations Between Chronotype, Genetic Susceptibility and Risk of Colorectal Cancer in UK Biobank
Source: J Epidemiol Glob Health. 2025 Apr 10;15(1):57. doi: 10.1007/s44197-025-00399-6 (PMC11985712; doi:10.1007/s44197-025-00399-6)
Supplement: Supplementary file 1 — Supplementary file1 (DOCX 47 KB) [file 44197_2025_399_MOESM1_ESM.docx]

| Table S1. Genetic instrumental variables used for genetic liability to chronotype | | | | | | | | | |  |
| --- | --- | --- | --- | --- | --- | --- | --- | --- | --- | --- |
| No | SNP | Chr | Position | EA | EAF | Beta_meta | SE_meta | Beta_23 | SE_23 |  |
| 1 | rs10916892 | 1 | 21201325 | T | 0.62 | -0.035 | 0.004 | -0.036 | 0.006 |  |
| 2 | rs11102807 | 1 | 115061584 | A | 0.54 | -0.022 | 0.004 | -0.019 | 0.006 |  |
| 3 | rs11165655 | 1 | 96959104 | A | 0.53 | -0.028 | 0.004 | -0.029 | 0.006 |  |
| 4 | rs11208844 | 1 | 66851147 | A | 0.14 | -0.029 | 0.005 | -0.032 | 0.008 |  |
| 5 | rs1144566 | 1 | 182569626 | T | 0.03 | 0.231 | 0.01 | 0.312 | 0.018 |  |
| 6 | rs115073088 | 1 | 174215858 | A | 0.98 | -0.076 | 0.011 | -0.077 | 0.018 |  |
| 7 | rs11588913 | 1 | 79963816 | A | 0.4 | -0.024 | 0.004 | -0.021 | 0.006 |  |
| 8 | rs12040629 | 1 | 77705365 | A | 0.16 | 0.073 | 0.005 | 0.083 | 0.008 |  |
| 9 | rs12065331 | 1 | 14507831 | T | 0.31 | -0.024 | 0.004 | -0.03 | 0.007 |  |
| 10 | rs12140153 | 1 | 62579891 | T | 0.09 | -0.06 | 0.007 | -0.068 | 0.012 |  |
| 11 | rs1221502 | 1 | 193276975 | A | 0.74 | 0.02 | 0.003 | 0.023 | 0.007 |  |
| 12 | rs17448682 | 1 | 15966713 | T | 0.23 | 0.035 | 0.004 | 0.035 | 0.007 |  |
| 13 | rs17575798 | 1 | 110086451 | A | 0.19 | -0.034 | 0.004 | -0.034 | 0.008 |  |
| 14 | rs4657983 | 1 | 195454557 | A | 0.65 | -0.025 | 0.004 | -0.031 | 0.006 |  |
| 15 | rs481214 | 1 | 93469865 | A | 0.61 | 0.023 | 0.004 | 0.02 | 0.006 |  |
| 16 | rs5016898 | 1 | 81672013 | T | 0.42 | -0.024 | 0.004 | -0.029 | 0.006 |  |
| 17 | rs61773390 | 1 | 7884525 | T | 0.19 | 0.066 | 0.005 | 0.077 | 0.008 |  |
| 18 | rs6429233 | 1 | 241137033 | A | 0.46 | 0.02 | 0.003 | 0.022 | 0.006 |  |
| 19 | rs6665637 | 1 | 153756083 | A | 0.28 | -0.02 | 0.003 | -0.027 | 0.007 |  |
| 20 | rs6690292 | 1 | 113188419 | T | 0.73 | -0.025 | 0.004 | -0.025 | 0.007 |  |
| 21 | rs72720396 | 1 | 91191582 | A | 0.77 | -0.042 | 0.004 | -0.037 | 0.007 |  |
| 22 | rs9436119 | 1 | 150467753 | A | 0.38 | 0.04 | 0.003 | 0.053 | 0.006 |  |
| 23 | rs975025 | 1 | 179338327 | T | 0.08 | -0.049 | 0.006 | -0.052 | 0.011 |  |
| 24 | rs10175975 | 2 | 59429807 | T | 0.19 | 0.025 | 0.004 | 0.016 | 0.008 |  |
| 25 | rs10520176 | 2 | 77217310 | T | 0.49 | 0.038 | 0.004 | 0.039 | 0.006 |  |
| 26 | rs1064213 | 2 | 198950240 | A | 0.48 | 0.044 | 0.004 | 0.065 | 0.006 |  |
| 27 | rs113851554 | 2 | 66750564 | T | 0.06 | -0.054 | 0.007 | -0.067 | 0.014 |  |
| 28 | rs11677484 | 2 | 191578172 | T | 0.26 | 0.023 | 0.004 | 0.018 | 0.007 |  |
| 29 | rs11678584 | 2 | 32563426 | A | 0.86 | -0.028 | 0.005 | -0.041 | 0.009 |  |
| 30 | rs11681299 | 2 | 88901732 | T | 0.28 | 0.024 | 0.004 | 0.035 | 0.007 |  |
| 31 | rs12464387 | 2 | 75445544 | A | 0.46 | -0.021 | 0.003 | -0.024 | 0.006 |  |
| 32 | rs12470914 | 2 | 50532840 | A | 0.1 | 0.053 | 0.006 | 0.069 | 0.01 |  |
| 33 | rs13004345 | 2 | 174037347 | T | 0.65 | -0.019 | 0.003 | -0.025 | 0.006 |  |
| 34 | rs13011556 | 2 | 4651923 | C | 0.76 | -0.029 | 0.004 | -0.025 | 0.007 |  |
| 35 | rs13414393 | 2 | 54275162 | T | 0.54 | -0.022 | 0.004 | -0.021 | 0.006 |  |
| 36 | rs17396357 | 2 | 48252311 | T | 0.38 | 0.021 | 0.004 | 0.032 | 0.006 |  |
| 37 | rs184033703 | 2 | 206956138 | A | 0.06 | 0.058 | 0.008 | 0.054 | 0.013 |  |
| 38 | rs2166559 | 2 | 149551658 | T | 0.86 | -0.033 | 0.005 | -0.025 | 0.009 |  |
| 39 | rs2706762 | 2 | 70488470 | T | 0.15 | -0.037 | 0.005 | -0.045 | 0.008 |  |
| 40 | rs28380327 | 2 | 144232491 | A | 0.63 | 0.04 | 0.004 | 0.052 | 0.006 |  |
| 41 | rs34509802 | 2 | 101591710 | A | 0.18 | 0.04 | 0.005 | 0.051 | 0.008 |  |
| 42 | rs359248 | 2 | 60477461 | T | 0.46 | -0.028 | 0.003 | -0.038 | 0.006 |  |
| 43 | rs4666682 | 2 | 186203743 | A | 0.18 | -0.025 | 0.004 | -0.029 | 0.008 |  |
| 44 | rs4672458 | 2 | 53736362 | T | 0.48 | -0.022 | 0.003 | -0.018 | 0.006 |  |
| 45 | rs62124718 | 2 | 12822995 | A | 0.9 | -0.045 | 0.006 | -0.057 | 0.01 |  |
| 46 | rs62182135 | 2 | 240267305 | A | 0.33 | -0.024 | 0.003 | -0.026 | 0.006 |  |
| 47 | rs6433478 | 2 | 175241482 | T | 0.46 | -0.025 | 0.004 | -0.035 | 0.006 |  |
| 48 | rs6544906 | 2 | 46863872 | A | 0.56 | 0.023 | 0.004 | 0.03 | 0.006 |  |
| 49 | rs6727752 | 2 | 76361783 | A | 0.36 | 0.026 | 0.004 | 0.023 | 0.007 |  |
| 50 | rs72796401 | 2 | 24180078 | A | 0.19 | 0.025 | 0.003 | 0.029 | 0.008 |  |
| 51 | rs747003 | 2 | 161916409 | T | 0.61 | 0.02 | 0.003 | 0.017 | 0.006 |  |
| 52 | rs75120545 | 2 | 44271496 | T | 0.03 | 0.086 | 0.01 | 0.085 | 0.018 |  |
| 53 | rs76064513 | 2 | 125438641 | T | 0.13 | 0.034 | 0.006 | 0.042 | 0.009 |  |
| 54 | rs77248969 | 2 | 136490731 | A | 0.11 | -0.033 | 0.006 | -0.034 | 0.009 |  |
| 55 | rs80271258 | 2 | 239311505 | T | 0.08 | -0.089 | 0.006 | -0.096 | 0.011 |  |
| 56 | rs812925 | 2 | 61680993 | C | 0.65 | -0.031 | 0.004 | -0.031 | 0.006 |  |
| 57 | rs848552 | 2 | 36700580 | C | 0.48 | -0.028 | 0.004 | -0.033 | 0.006 |  |
| 58 | rs111261826 | 3 | 7189617 | A | 0.68 | -0.028 | 0.004 | -0.031 | 0.006 |  |
| 59 | rs111867612 | 3 | 157721819 | A | 0.1 | -0.032 | 0.006 | -0.033 | 0.01 |  |
| 60 | rs112201801 | 3 | 82591379 | T | 0.93 | -0.085 | 0.013 | -0.085 | 0.013 |  |
| 61 | rs114848860 | 3 | 36859494 | A | 0.97 | -0.077 | 0.01 | -0.095 | 0.021 |  |
| 62 | rs12636669 | 3 | 50003323 | T | 0.08 | 0.057 | 0.006 | 0.071 | 0.011 |  |
| 63 | rs13065394 | 3 | 132971327 | T | 0.29 | -0.027 | 0.004 | -0.024 | 0.007 |  |
| 64 | rs1398346 | 3 | 110271943 | T | 0.87 | 0.026 | 0.005 | 0.032 | 0.009 |  |
| 65 | rs1449403 | 3 | 85591467 | A | 0.12 | 0.042 | 0.006 | 0.055 | 0.009 |  |
| 66 | rs1468945 | 3 | 185990392 | A | 0.79 | -0.036 | 0.004 | -0.035 | 0.007 |  |
| 67 | rs149611468 | 3 | 8817423 | T | 0.99 | 0.143 | 0.018 | 0.174 | 0.032 |  |
| 68 | rs1599374 | 3 | 160891727 | A | 0.52 | 0.031 | 0.004 | 0.037 | 0.006 |  |
| 69 | rs17007397 | 3 | 70594975 | C | 0.58 | 0.023 | 0.004 | 0.026 | 0.006 |  |
| 70 | rs1800828 | 3 | 113891549 | C | 0.75 | 0.026 | 0.004 | 0.016 | 0.007 |  |
| 71 | rs2362775 | 3 | 24924421 | T | 0.53 | -0.022 | 0.004 | -0.008 | 0.006 |  |
| 72 | rs301218 | 3 | 176096919 | A | 0.39 | -0.024 | 0.004 | -0.032 | 0.006 |  |
| 73 | rs34967119 | 3 | 104778430 | A | 0.5 | 0.02 | 0.003 | 0.023 | 0.006 |  |
| 74 | rs35346733 | 3 | 2521322 | A | 0.19 | -0.032 | 0.005 | -0.032 | 0.008 |  |
| 75 | rs3850174 | 3 | 172364093 | A | 0.26 | -0.035 | 0.004 | -0.039 | 0.007 |  |
| 76 | rs6440833 | 3 | 152646244 | A | 0.46 | 0.021 | 0.003 | 0.029 | 0.006 |  |
| 77 | rs72950188 | 3 | 116103275 | T | 0.92 | 0.045 | 0.007 | 0.053 | 0.011 |  |
| 78 | rs72966564 | 3 | 123149816 | T | 0.25 | -0.023 | 0.004 | -0.019 | 0.007 |  |
| 79 | rs73050286 | 3 | 23224684 | T | 0.78 | 0.03 | 0.004 | 0.03 | 0.007 |  |
| 80 | rs7429614 | 3 | 77205438 | T | 0.42 | 0.035 | 0.004 | 0.046 | 0.006 |  |
| 81 | rs7626335 | 3 | 71575177 | A | 0.33 | -0.029 | 0.004 | -0.041 | 0.006 |  |
| 82 | rs7649164 | 3 | 150788032 | T | 0.57 | 0.021 | 0.004 | 0.024 | 0.006 |  |
| 83 | rs9817910 | 3 | 18246870 | A | 0.56 | -0.022 | 0.003 | -0.016 | 0.006 |  |
| 84 | rs9836621 | 3 | 182096311 | T | 0.52 | -0.028 | 0.004 | -0.04 | 0.006 |  |
| 85 | rs1502249 | 4 | 27495379 | A | 0.52 | 0.017 | 0.003 | 0.022 | 0.006 |  |
| 86 | rs17455138 | 4 | 130903511 | T | 0.77 | 0.031 | 0.005 | 0.041 | 0.007 |  |
| 87 | rs2850979 | 4 | 102094764 | T | 0.76 | -0.023 | 0.004 | -0.022 | 0.007 |  |
| 88 | rs3796618 | 4 | 1349602 | A | 0.53 | -0.023 | 0.004 | -0.02 | 0.006 |  |
| 89 | rs4241964 | 4 | 137053959 | T | 0.52 | -0.029 | 0.003 | -0.023 | 0.006 |  |
| 90 | rs4690085 | 4 | 2697300 | A | 0.53 | -0.019 | 0.003 | -0.015 | 0.006 |  |
| 91 | rs4698678 | 4 | 18260776 | C | 0.28 | 0.031 | 0.004 | 0.038 | 0.007 |  |
| 92 | rs4860734 | 4 | 67096904 | A | 0.29 | 0.02 | 0.003 | 0.024 | 0.007 |  |
| 93 | rs6816922 | 4 | 80206272 | A | 0.54 | -0.02 | 0.004 | -0.022 | 0.006 |  |
| 94 | rs6838677 | 4 | 66520667 | A | 0.67 | -0.021 | 0.004 | -0.022 | 0.006 |  |
| 95 | rs6846730 | 4 | 83279041 | T | 0.24 | -0.032 | 0.004 | -0.029 | 0.007 |  |
| 96 | rs72729847 | 4 | 147296930 | T | 0.8 | -0.03 | 0.005 | -0.036 | 0.007 |  |
| 97 | rs7700110 | 4 | 114439894 | A | 0.26 | 0.024 | 0.004 | 0.03 | 0.007 |  |
| 98 | rs938836 | 4 | 139939653 | A | 0.47 | -0.021 | 0.003 | -0.027 | 0.006 |  |
| 99 | rs9991917 | 4 | 132512118 | A | 0.19 | 0.045 | 0.008 | 0.045 | 0.008 |  |
| 100 | rs9997394 | 4 | 163704083 | A | 0.29 | -0.025 | 0.004 | -0.031 | 0.007 |  |
| 101 | rs10058356 | 5 | 35220404 | T | 0.7 | -0.021 | 0.003 | -0.018 | 0.006 |  |
| 102 | rs12518401 | 5 | 173539588 | A | 0.38 | -0.024 | 0.004 | -0.034 | 0.006 |  |
| 103 | rs13172141 | 5 | 122990902 | A | 0.57 | 0.022 | 0.004 | 0.028 | 0.006 |  |
| 104 | rs1559253 | 5 | 106657015 | A | 0.36 | 0.022 | 0.004 | 0.036 | 0.006 |  |
| 105 | rs2901796 | 5 | 163330708 | A | 0.4 | 0.025 | 0.004 | 0.026 | 0.006 |  |
| 106 | rs42210 | 5 | 166408788 | C | 0.71 | -0.029 | 0.005 | -0.031 | 0.007 |  |
| 107 | rs4269995 | 5 | 87701223 | T | 0.25 | -0.034 | 0.004 | -0.047 | 0.007 |  |
| 108 | rs465670 | 5 | 176877624 | T | 0.54 | 0.024 | 0.004 | 0.023 | 0.006 |  |
| 109 | rs67988891 | 5 | 152204741 | C | 0.68 | -0.036 | 0.004 | -0.031 | 0.006 |  |
| 110 | rs7701529 | 5 | 63861475 | A | 0.24 | -0.03 | 0.004 | -0.029 | 0.007 |  |
| 111 | rs7721608 | 5 | 76581258 | T | 0.46 | 0.02 | 0.003 | 0.03 | 0.006 |  |
| 112 | rs7735794 | 5 | 175339984 | A | 0.22 | 0.034 | 0.006 | NA | NA |  |
| 113 | rs77960 | 5 | 103964585 | A | 0.33 | 0.022 | 0.003 | 0.016 | 0.006 |  |
| 114 | rs11154718 | 6 | 99592404 | T | 0.43 | -0.023 | 0.004 | -0.027 | 0.006 |  |
| 115 | rs12195792 | 6 | 98705295 | A | 0.27 | 0.034 | 0.004 | 0.047 | 0.007 |  |
| 116 | rs12206814 | 6 | 41517457 | C | 0.49 | 0.025 | 0.004 | 0.017 | 0.007 |  |
| 117 | rs1811899 | 6 | 14878060 | T | 0.79 | -0.03 | 0.005 | -0.037 | 0.007 |  |
| 118 | rs1931814 | 6 | 62589167 | A | 0.48 | 0.026 | 0.004 | 0.031 | 0.006 |  |
| 119 | rs2050185 | 6 | 147936781 | A | 0.62 | 0.022 | 0.004 | 0.018 | 0.006 |  |
| 120 | rs2396004 | 6 | 43355851 | A | 0.44 | 0.021 | 0.004 | 0.025 | 0.006 |  |
| 121 | rs2653349 | 6 | 55142337 | A | 0.21 | 0.066 | 0.004 | 0.074 | 0.008 |  |
| 122 | rs2881955 | 6 | 72479263 | T | 0.28 | 0.027 | 0.004 | 0.028 | 0.007 |  |
| 123 | rs3857599 | 6 | 50938247 | A | 0.17 | 0.032 | 0.005 | 0.019 | 0.008 |  |
| 124 | rs3923809 | 6 | 38440970 | A | 0.69 | -0.022 | 0.004 | -0.029 | 0.006 |  |
| 125 | rs4535583 | 6 | 115699280 | T | 0.7 | 0.021 | 0.004 | 0.027 | 0.007 |  |
| 126 | rs486416 | 6 | 31856070 | A | 0.65 | -0.02 | 0.003 | -0.02 | 0.006 |  |
| 127 | rs60616179 | 6 | 110244765 | A | 0.94 | 0.051 | 0.008 | 0.031 | 0.013 |  |
| 128 | rs766406 | 6 | 26319588 | T | 0.64 | -0.024 | 0.004 | -0.034 | 0.006 |  |
| 129 | rs9347926 | 6 | 165195547 | A | 0.44 | 0.026 | 0.003 | 0.032 | 0.006 |  |
| 130 | rs9348050 | 6 | 166263488 | T | 0.49 | 0.022 | 0.003 | 0.019 | 0.006 |  |
| 131 | rs9381812 | 6 | 13183998 | A | 0.71 | -0.05 | 0.004 | -0.07 | 0.007 |  |
| 132 | rs9394154 | 6 | 11574374 | C | 0.44 | -0.022 | 0.003 | -0.019 | 0.006 |  |
| 133 | rs9465253 | 6 | 19102247 | T | 0.28 | 0.023 | 0.004 | 0.026 | 0.007 |  |
| 134 | rs9479402 | 6 | 153135339 | T | 0.99 | -0.219 | 0.017 | -0.272 | 0.027 |  |
| 135 | rs9496623 | 6 | 143751625 | A | 0.73 | -0.024 | 0.004 | -0.023 | 0.007 |  |
| 136 | rs10237162 | 7 | 24085405 | T | 0.72 | 0.037 | 0.004 | 0.049 | 0.007 |  |
| 137 | rs10254050 | 7 | 96468077 | C | 0.19 | -0.058 | 0.005 | -0.076 | 0.008 |  |
| 138 | rs10951325 | 7 | 32265545 | T | 0.63 | 0.034 | 0.004 | 0.037 | 0.006 |  |
| 139 | rs113161209 | 7 | 148564367 | A | 0.08 | 0.044 | 0.008 | 0.039 | 0.011 |  |
| 140 | rs17302081 | 7 | 115673079 | T | 0.44 | 0.022 | 0.004 | 0.014 | 0.006 |  |
| 141 | rs2072413 | 7 | 150647969 | T | 0.26 | -0.021 | 0.004 | -0.024 | 0.007 |  |
| 142 | rs2944831 | 7 | 71779635 | A | 0.29 | 0.025 | 0.004 | 0.025 | 0.007 |  |
| 143 | rs3807651 | 7 | 77823771 | A | 0.49 | 0.025 | 0.004 | 0.022 | 0.006 |  |
| 144 | rs4027217 | 7 | 14093914 | A | 0.22 | -0.026 | 0.005 | -0.024 | 0.007 |  |
| 145 | rs4236237 | 7 | 69936477 | A | 0.6 | -0.024 | 0.004 | -0.031 | 0.006 |  |
| 146 | rs4729854 | 7 | 102383663 | A | 0.47 | -0.049 | 0.004 | -0.056 | 0.007 |  |
| 147 | rs62465218 | 7 | 132294312 | A | 0.15 | -0.027 | 0.005 | -0.039 | 0.009 |  |
| 148 | rs6958557 | 7 | 133585794 | T | 0.61 | 0.026 | 0.004 | 0.035 | 0.006 |  |
| 149 | rs6967481 | 7 | 50642701 | T | 0.5 | 0.032 | 0.003 | 0.036 | 0.006 |  |
| 150 | rs6968240 | 7 | 121942674 | A | 0.42 | 0.022 | 0.003 | 0.033 | 0.006 |  |
| 151 | rs10109566 | 8 | 59800446 | A | 0.48 | -0.022 | 0.004 | -0.022 | 0.006 |  |
| 152 | rs16939162 | 8 | 76653156 | A | 0.83 | 0.038 | 0.005 | 0.033 | 0.008 |  |
| 153 | rs187028 | 8 | 73459513 | A | 0.31 | -0.022 | 0.003 | -0.028 | 0.006 |  |
| 154 | rs1871729 | 8 | 136223702 | A | 0.68 | -0.023 | 0.004 | -0.026 | 0.006 |  |
| 155 | rs2322605 | 8 | 27164449 | A | 0.47 | -0.022 | 0.004 | -0.025 | 0.006 |  |
| 156 | rs2737245 | 8 | 116658583 | T | 0.27 | 0.034 | 0.004 | 0.035 | 0.007 |  |
| 157 | rs2979139 | 8 | 8268313 | A | 0.5 | -0.027 | 0.003 | -0.03 | 0.006 |  |
| 158 | rs3100052 | 8 | 101967139 | A | 0.39 | 0.025 | 0.004 | 0.021 | 0.006 |  |
| 159 | rs34054660 | 8 | 65015659 | A | 0.57 | 0.025 | 0.004 | 0.033 | 0.006 |  |
| 160 | rs35524253 | 8 | 4823608 | A | 0.35 | 0.034 | 0.004 | 0.047 | 0.006 |  |
| 161 | rs62479736 | 8 | 3654320 | T | 0.29 | 0.024 | 0.004 | 0.026 | 0.007 |  |
| 162 | rs6468316 | 8 | 35237788 | T | 0.47 | -0.02 | 0.003 | -0.015 | 0.006 |  |
| 163 | rs6988733 | 8 | 91535686 | T | 0.35 | 0.023 | 0.004 | 0.025 | 0.006 |  |
| 164 | rs6993892 | 8 | 33729200 | T | 0.62 | -0.035 | 0.004 | -0.037 | 0.006 |  |
| 165 | rs7006885 | 8 | 93283578 | A | 0.29 | 0.03 | 0.004 | 0.05 | 0.007 |  |
| 166 | rs71523448 | 8 | 31817493 | C | 0.08 | -0.05 | 0.007 | -0.063 | 0.012 |  |
| 167 | rs7845620 | 8 | 53129069 | A | 0.83 | -0.043 | 0.005 | -0.058 | 0.008 |  |
| 168 | rs10759208 | 9 | 109806199 | T | 0.61 | -0.025 | 0.004 | -0.026 | 0.006 |  |
| 169 | rs10818834 | 9 | 126317324 | T | 0.73 | 0.03 | 0.004 | 0.026 | 0.007 |  |
| 170 | rs10988239 | 9 | 131943440 | T | 0.51 | -0.021 | 0.003 | -0.016 | 0.006 |  |
| 171 | rs11788633 | 9 | 116767656 | C | 0.65 | 0.02 | 0.003 | 0.029 | 0.006 |  |
| 172 | rs12378543 | 9 | 83196097 | T | 0.38 | -0.023 | 0.004 | -0.022 | 0.006 |  |
| 173 | rs12380242 | 9 | 139310187 | T | 0.51 | -0.021 | 0.003 | -0.016 | 0.006 |  |
| 174 | rs2844016 | 9 | 24582747 | T | 0.29 | 0.027 | 0.004 | 0.031 | 0.007 |  |
| 175 | rs295268 | 9 | 86429305 | T | 0.74 | -0.031 | 0.005 | -0.026 | 0.007 |  |
| 176 | rs308521 | 9 | 37367094 | T | 0.6 | 0.028 | 0.003 | 0.031 | 0.006 |  |
| 177 | rs3138490 | 9 | 92219000 | A | 0.52 | 0.024 | 0.004 | 0.023 | 0.006 |  |
| 178 | rs4878734 | 9 | 38010085 | A | 0.51 | 0.022 | 0.004 | 0.016 | 0.006 |  |
| 179 | rs555784 | 9 | 85318704 | A | 0.38 | -0.025 | 0.004 | -0.029 | 0.006 |  |
| 180 | rs62553781 | 9 | 76679777 | T | 0.03 | -0.069 | 0.009 | -0.06 | 0.018 |  |
| 181 | rs6477309 | 9 | 8450638 | T | 0.67 | 0.031 | 0.004 | 0.035 | 0.006 |  |
| 182 | rs6560218 | 9 | 74245426 | T | 0.52 | -0.022 | 0.004 | -0.021 | 0.006 |  |
| 183 | rs10762434 | 10 | 73044413 | C | 0.78 | 0.025 | 0.004 | 0.033 | 0.007 |  |
| 184 | rs10830107 | 10 | 129304075 | A | 0.79 | 0.028 | 0.005 | 0.031 | 0.007 |  |
| 185 | rs11200159 | 10 | 123553392 | A | 0.66 | -0.023 | 0.004 | -0.023 | 0.006 |  |
| 186 | rs1163238 | 10 | 104943993 | A | 0.39 | -0.024 | 0.004 | -0.018 | 0.006 |  |
| 187 | rs12249410 | 10 | 64301941 | T | 0.11 | -0.034 | 0.006 | -0.031 | 0.01 |  |
| 188 | rs12771973 | 10 | 133749294 | A | 0.25 | -0.022 | 0.004 | -0.028 | 0.007 |  |
| 189 | rs17712705 | 10 | 69623271 | A | 0.33 | -0.025 | 0.004 | -0.029 | 0.006 |  |
| 190 | rs2298117 | 10 | 70346740 | T | 0.45 | -0.023 | 0.004 | -0.018 | 0.006 |  |
| 191 | rs3808964 | 10 | 125426627 | T | 0.63 | 0.02 | 0.003 | 0.015 | 0.006 |  |
| 192 | rs497338 | 10 | 804315 | T | 0.29 | 0.027 | 0.004 | 0.029 | 0.006 |  |
| 193 | rs61875203 | 10 | 93888810 | T | 0.28 | 0.026 | 0.004 | 0.027 | 0.007 |  |
| 194 | rs66617308 | 10 | 56699338 | T | 0.67 | 0.018 | 0.003 | 0.017 | 0.006 |  |
| 195 | rs76518095 | 10 | 131149976 | T | 0.08 | 0.04 | 0.007 | 0.046 | 0.012 |  |
| 196 | rs7900191 | 10 | 119145774 | T | 0.4 | -0.019 | 0.003 | -0.018 | 0.006 |  |
| 197 | rs9416744 | 10 | 60567937 | A | 0.26 | 0.034 | 0.004 | 0.044 | 0.007 |  |
| 198 | rs9664044 | 10 | 126710791 | T | 0.23 | -0.027 | 0.004 | -0.025 | 0.007 |  |
| 199 | rs10742179 | 11 | 27650524 | A | 0.26 | 0.035 | 0.004 | 0.038 | 0.007 |  |
| 200 | rs10832648 | 11 | 16618307 | A | 0.2 | -0.031 | 0.004 | -0.03 | 0.007 |  |
| 201 | rs10838687 | 11 | 47312892 | T | 0.79 | 0.035 | 0.005 | 0.045 | 0.007 |  |
| 202 | rs11032362 | 11 | 33759092 | A | 0.09 | 0.07 | 0.006 | 0.079 | 0.01 |  |
| 203 | rs1278402 | 11 | 82972097 | A | 0.74 | 0.028 | 0.005 | 0.038 | 0.007 |  |
| 204 | rs12808544 | 11 | 58373221 | A | 0.24 | -0.035 | 0.004 | -0.034 | 0.007 |  |
| 205 | rs1508608 | 11 | 92893825 | A | 0.32 | 0.028 | 0.004 | 0.04 | 0.006 |  |
| 206 | rs17577073 | 11 | 99152801 | A | 0.56 | 0.025 | 0.004 | 0.028 | 0.006 |  |
| 207 | rs2514214 | 11 | 113395329 | A | 0.27 | 0.027 | 0.004 | 0.026 | 0.007 |  |
| 208 | rs3867239 | 11 | 122093090 | A | 0.38 | 0.026 | 0.004 | 0.032 | 0.006 |  |
| 209 | rs4121878 | 11 | 95120372 | C | 0.5 | 0.022 | 0.004 | 0.024 | 0.006 |  |
| 210 | rs4923541 | 11 | 28479535 | T | 0.51 | 0.025 | 0.004 | 0.028 | 0.006 |  |
| 211 | rs4936290 | 11 | 114009255 | A | 0.66 | -0.023 | 0.003 | -0.019 | 0.007 |  |
| 212 | rs621421 | 11 | 30405914 | T | 0.63 | -0.027 | 0.003 | -0.029 | 0.006 |  |
| 213 | rs662094 | 11 | 66342691 | A | 0.49 | 0.028 | 0.004 | 0.036 | 0.006 |  |
| 214 | rs7111582 | 11 | 43893222 | A | 0.9 | -0.039 | 0.005 | -0.048 | 0.01 |  |
| 215 | rs74357745 | 11 | 122811822 | A | 0.88 | 0.031 | 0.005 | 0.021 | 0.009 |  |
| 216 | rs7943634 | 11 | 126734319 | T | 0.31 | -0.024 | 0.004 | -0.02 | 0.006 |  |
| 217 | rs10877962 | 12 | 63520912 | T | 0.41 | 0.036 | 0.004 | 0.049 | 0.006 |  |
| 218 | rs11611435 | 12 | 24089322 | T | 0.56 | 0.028 | 0.004 | 0.022 | 0.006 |  |
| 219 | rs12298405 | 12 | 17015267 | T | 0.33 | -0.023 | 0.004 | -0.024 | 0.006 |  |
| 220 | rs1799464 | 12 | 16286082 | A | 0.29 | -0.02 | 0.004 | -0.025 | 0.007 |  |
| 221 | rs1843888 | 12 | 38737310 | A | 0.54 | 0.051 | 0.004 | 0.06 | 0.006 |  |
| 222 | rs2433634 | 12 | 23060363 | A | 0.72 | -0.027 | 0.004 | -0.028 | 0.007 |  |
| 223 | rs247929 | 12 | 46294908 | C | 0.51 | 0.031 | 0.004 | 0.036 | 0.006 |  |
| 224 | rs3782860 | 12 | 361996 | T | 0.54 | 0.025 | 0.004 | 0.029 | 0.006 |  |
| 225 | rs3955311 | 12 | 114343818 | T | 0.15 | 0.026 | 0.005 | 0.031 | 0.008 |  |
| 226 | rs711098 | 12 | 77976559 | A | 0.4 | 0.022 | 0.004 | 0.033 | 0.006 |  |
| 227 | rs7298532 | 12 | 112510404 | T | 0.72 | 0.027 | 0.004 | 0.025 | 0.007 |  |
| 228 | rs7299922 | 12 | 54702519 | A | 0.64 | 0.024 | 0.004 | 0.023 | 0.006 |  |
| 229 | rs7304278 | 12 | 106989915 | A | 0.28 | -0.029 | 0.004 | -0.03 | 0.007 |  |
| 230 | rs7959983 | 12 | 90452978 | T | 0.6 | -0.03 | 0.004 | -0.028 | 0.006 |  |
| 231 | rs7975791 | 12 | 49413486 | T | 0.04 | 0.051 | 0.009 | 0.054 | 0.016 |  |
| 232 | rs80097534 | 12 | 121029604 | T | 0.1 | -0.036 | 0.006 | -0.024 | 0.011 |  |
| 233 | rs1163628 | 13 | 112226420 | A | 0.86 | -0.029 | 0.005 | -0.033 | 0.008 |  |
| 234 | rs1886205 | 13 | 94062095 | A | 0.76 | 0.029 | 0.004 | 0.034 | 0.007 |  |
| 235 | rs2593487 | 13 | 69903058 | A | 0.34 | -0.029 | 0.004 | -0.034 | 0.006 |  |
| 236 | rs3815983 | 13 | 109779906 | T | 0.36 | -0.022 | 0.003 | -0.025 | 0.006 |  |
| 237 | rs45597035 | 13 | 73649152 | A | 0.65 | -0.022 | 0.004 | -0.023 | 0.006 |  |
| 238 | rs495593 | 13 | 72919800 | A | 0.74 | 0.023 | 0.004 | 0.032 | 0.007 |  |
| 239 | rs9558942 | 13 | 107700218 | T | 0.67 | -0.019 | 0.003 | -0.032 | 0.006 |  |
| 240 | rs9571526 | 13 | 66590868 | T | 0.77 | -0.027 | 0.005 | -0.032 | 0.007 |  |
| 241 | rs9573980 | 13 | 77590741 | A | 0.97 | 0.127 | 0.01 | 0.15 | 0.017 |  |
| 242 | rs9597241 | 13 | 56281271 | A | 0.81 | 0.033 | 0.004 | 0.037 | 0.008 |  |
| 243 | rs11845599 | 14 | 101016824 | A | 0.64 | -0.027 | 0.004 | -0.034 | 0.006 |  |
| 244 | rs2878172 | 14 | 55373670 | A | 0.57 | -0.021 | 0.004 | -0.03 | 0.006 |  |
| 245 | rs2978382 | 14 | 64769074 | T | 0.59 | 0.023 | 0.004 | 0.021 | 0.006 |  |
| 246 | rs4550384 | 14 | 85350142 | T | 0.76 | 0.024 | 0.004 | 0.028 | 0.007 |  |
| 247 | rs4903203 | 14 | 74660508 | A | 0.32 | 0.025 | 0.004 | 0.027 | 0.006 |  |
| 248 | rs61990287 | 14 | 42069889 | A | 0.27 | 0.025 | 0.004 | 0.027 | 0.007 |  |
| 249 | rs6573308 | 14 | 60806976 | T | 0.39 | 0.025 | 0.004 | 0.026 | 0.006 |  |
| 250 | rs710284 | 14 | 98532540 | T | 0.58 | 0.022 | 0.004 | 0.025 | 0.006 |  |
| 251 | rs7143933 | 14 | 62460219 | T | 0.26 | 0.025 | 0.004 | 0.034 | 0.007 |  |
| 252 | rs962961 | 14 | 57281154 | T | 0.33 | -0.022 | 0.003 | -0.025 | 0.006 |  |
| 253 | rs12442008 | 15 | 53725112 | T | 0.26 | 0.029 | 0.004 | 0.035 | 0.007 |  |
| 254 | rs12442674 | 15 | 96907819 | A | 0.73 | 0.023 | 0.004 | 0.04 | 0.008 |  |
| 255 | rs1873958 | 15 | 101147726 | A | 0.41 | 0.028 | 0.003 | 0.041 | 0.006 |  |
| 256 | rs4775086 | 15 | 58969292 | A | 0.24 | -0.027 | 0.005 | -0.03 | 0.007 |  |
| 257 | rs59986227 | 15 | 48009263 | C | 0.75 | -0.031 | 0.004 | -0.029 | 0.007 |  |
| 258 | rs11641239 | 16 | 23124193 | T | 0.29 | 0.023 | 0.004 | 0.017 | 0.007 |  |
| 259 | rs12445235 | 16 | 8195278 | C | 0.41 | -0.021 | 0.004 | -0.024 | 0.006 |  |
| 260 | rs12927162 | 16 | 52684916 | A | 0.73 | 0.056 | 0.004 | 0.066 | 0.007 |  |
| 261 | rs1421085 | 16 | 53800954 | T | 0.59 | -0.042 | 0.003 | -0.044 | 0.006 |  |
| 262 | rs17604349 | 16 | 72210865 | A | 0.18 | -0.037 | 0.004 | -0.044 | 0.008 |  |
| 263 | rs2304467 | 16 | 8988777 | C | 0.61 | -0.024 | 0.004 | -0.02 | 0.006 |  |
| 264 | rs2550298 | 16 | 56367969 | T | 0.38 | -0.04 | 0.004 | -0.043 | 0.006 |  |
| 265 | rs4785296 | 16 | 49467234 | C | 0.23 | 0.026 | 0.004 | 0.029 | 0.007 |  |
| 266 | rs7203707 | 16 | 24518569 | A | 0.52 | -0.02 | 0.003 | -0.016 | 0.006 |  |
| 267 | rs72773411 | 16 | 728514 | A | 0.15 | 0.029 | 0.005 | 0.035 | 0.009 |  |
| 268 | rs72790386 | 16 | 68136932 | T | 0.03 | 0.06 | 0.011 | 0.071 | 0.017 |  |
| 269 | rs8044054 | 16 | 60628436 | T | 0.39 | 0.031 | 0.004 | 0.031 | 0.006 |  |
| 270 | rs1061032 | 17 | 8064083 | T | 0.09 | 0.064 | 0.006 | 0.065 | 0.01 |  |
| 271 | rs11545787 | 17 | 17398278 | A | 0.25 | -0.05 | 0.004 | -0.07 | 0.007 |  |
| 272 | rs12051 | 17 | 46103760 | A | 0.61 | -0.026 | 0.004 | -0.033 | 0.006 |  |
| 273 | rs12600452 | 17 | 45054564 | A | 0.2 | 0.026 | 0.004 | 0.034 | 0.008 |  |
| 274 | rs12950382 | 17 | 30603994 | A | 0.72 | 0.023 | 0.004 | 0.027 | 0.007 |  |
| 275 | rs2011528 | 17 | 33980566 | T | 0.83 | -0.033 | 0.005 | -0.029 | 0.008 |  |
| 276 | rs2916148 | 17 | 65482109 | A | 0.45 | 0.028 | 0.004 | 0.033 | 0.006 |  |
| 277 | rs3760381 | 17 | 43047083 | A | 0.25 | 0.027 | 0.004 | 0.03 | 0.007 |  |
| 278 | rs412000 | 17 | 56709058 | C | 0.56 | -0.022 | 0.004 | -0.025 | 0.006 |  |
| 279 | rs4365329 | 17 | 31625887 | A | 0.54 | -0.019 | 0.003 | -0.025 | 0.006 |  |
| 280 | rs55846845 | 17 | 50092201 | A | 0.52 | -0.021 | 0.003 | -0.021 | 0.006 |  |
| 281 | rs58681483 | 17 | 57934654 | A | 0.92 | 0.035 | 0.006 | 0.055 | 0.01 |  |
| 282 | rs72829706 | 17 | 54173733 | A | 0.96 | 0.056 | 0.009 | 0.052 | 0.015 |  |
| 283 | rs72841368 | 17 | 61391114 | A | 0.81 | -0.03 | 0.004 | -0.03 | 0.008 |  |
| 284 | rs8072058 | 17 | 55734198 | A | 0.78 | -0.028 | 0.005 | -0.031 | 0.007 |  |
| 285 | rs1013987 | 18 | 22630836 | T | 0.4 | -0.029 | 0.004 | -0.022 | 0.006 |  |
| 286 | rs1025601 | 18 | 73056278 | T | 0.39 | -0.022 | 0.004 | -0.016 | 0.006 |  |
| 287 | rs11152350 | 18 | 60240352 | A | 0.47 | -0.028 | 0.004 | -0.029 | 0.006 |  |
| 288 | rs12969848 | 18 | 38152835 | T | 0.53 | 0.036 | 0.004 | 0.036 | 0.006 |  |
| 289 | rs1788784 | 18 | 21159630 | A | 0.34 | -0.027 | 0.004 | -0.043 | 0.006 |  |
| 290 | rs2580160 | 18 | 1816036 | A | 0.56 | 0.028 | 0.004 | 0.024 | 0.006 |  |
| 291 | rs34329963 | 18 | 64526233 | T | 0.11 | -0.032 | 0.005 | -0.039 | 0.009 |  |
| 292 | rs4419127 | 18 | 31663654 | A | 0.66 | 0.044 | 0.004 | 0.06 | 0.006 |  |
| 293 | rs4800998 | 18 | 53429655 | A | 0.18 | 0.039 | 0.005 | 0.057 | 0.008 |  |
| 294 | rs62082402 | 18 | 5186566 | T | 0.19 | 0.05 | 0.005 | 0.06 | 0.011 |  |
| 295 | rs9950528 | 18 | 35762461 | A | 0.65 | -0.024 | 0.004 | -0.025 | 0.006 |  |
| 296 | rs9956387 | 18 | 44773382 | A | 0.5 | -0.02 | 0.003 | -0.018 | 0.006 |  |
| 297 | rs9964420 | 18 | 56824041 | A | 0.3 | -0.049 | 0.004 | -0.064 | 0.007 |  |
| 298 | rs10402849 | 19 | 2695661 | T | 0.2 | 0.026 | 0.004 | 0.02 | 0.008 |  |
| 299 | rs11670534 | 19 | 47003906 | T | 0.16 | -0.031 | 0.005 | -0.026 | 0.008 |  |
| 300 | rs36055559 | 19 | 5799433 | A | 0.17 | -0.036 | 0.005 | -0.045 | 0.01 |  |
| 301 | rs56113850 | 19 | 41353107 | T | 0.42 | -0.023 | 0.004 | -0.031 | 0.006 |  |
| 302 | rs58876439 | 19 | 42600984 | A | 0.07 | 0.047 | 0.007 | 0.053 | 0.012 |  |
| 303 | rs7248205 | 19 | 10770305 | T | 0.6 | 0.027 | 0.004 | 0.032 | 0.006 |  |
| 304 | rs73026775 | 19 | 31052954 | A | 0.12 | -0.034 | 0.006 | -0.041 | 0.01 |  |
| 305 | rs9636202 | 19 | 18449238 | A | 0.27 | -0.026 | 0.004 | -0.023 | 0.007 |  |
| 306 | rs1474754 | 20 | 20077178 | A | 0.26 | -0.021 | 0.004 | -0.029 | 0.007 |  |
| 307 | rs1737893 | 20 | 31051699 | T | 0.38 | -0.025 | 0.004 | -0.028 | 0.006 |  |
| 308 | rs2072727 | 20 | 43538733 | T | 0.43 | 0.028 | 0.003 | 0.033 | 0.006 |  |
| 309 | rs57236847 | 20 | 44668401 | C | 0.6 | 0.027 | 0.004 | 0.03 | 0.006 |  |
| 310 | rs6047481 | 20 | 21539564 | A | 0.67 | 0.025 | 0.004 | 0.025 | 0.006 |  |
| 311 | rs6131805 | 20 | 16222093 | T | 0.4 | 0.026 | 0.004 | 0.026 | 0.006 |  |
| 312 | rs6131942 | 20 | 17348608 | A | 0.42 | -0.026 | 0.003 | -0.033 | 0.006 |  |
| 313 | rs139911 | 22 | 40704052 | T | 0.57 | -0.034 | 0.004 | -0.024 | 0.006 |  |
| 314 | rs28459838 | 22 | 35846168 | T | 0.24 | 0.027 | 0.004 | 0.032 | 0.008 |  |
| 315 | rs6007594 | 22 | 45728370 | A | 0.26 | -0.025 | 0.004 | -0.025 | 0.007 |  |
| 316 | rs695459 | 22 | 28848278 | T | 0.39 | -0.022 | 0.004 | -0.019 | 0.006 |  |
| SNP, a specific position in the genome with potential variation between individuals; Chr, chromosome where the SNP is located; Position, the exact location of the SNP on the chromosome; EA, stands for Effect Allele, the allele believed to impact the analysis; EAF, stands for Effect Allele Frequency, the frequency of the effect allele in the study population; Beta_meta, stands for the effect size in the meta-analysis, a negative value indicates a decreased risk, while a positive value indicates an increased risk; SE_meta, stands for Standard Error of the effect size, indicating the precision of the Beta value. | | | | | | | | | |  |
|  |  |  |  |  |  |  |  |  |  |  |
|  |  |  |  |  |  |  |  |  |  |  |
|  |  |  |  |  |  |  |  |  |  |  |
|  |  |  |  |  |  |  |  |  |  |  |
|  |  |  |  |  |  |  |  |  |  |  |
|  |  |  |  |  |  |  |  |  |  |  |
