# Supplementary material for: Associations Between Chronotype, Genetic Susceptibility and Risk of Colorectal Cancer in UK Biobank
Source: J Epidemiol Glob Health. 2025 Apr 10;15(1):57. doi: 10.1007/s44197-025-00399-6 (PMC11985712; doi:10.1007/s44197-025-00399-6)
Supplement: Supplementary file 2 — Supplementary file2 (DOCX 14 KB) [file 44197_2025_399_MOESM2_ESM.docx]

| Table S2. Data fields and information on variables in the UK Biobank cohort involved in this study | | |
| --- | --- | --- |
| Category | Data field | Description |
| Demographic factors |  |  |
| Age | 21022 | Age at recruitment |
|  | 53 | Date of attending assessment center |
| Date of death | 40000 | Date of death |
| Sex | 31 | Sex |
| Socioeconomic status | 22189 | Townsend deprivation index at recruitment |
| Lifestyle factors |  |  |
| BMI | 21001 | Body mass index (BMI) |
| Alcohol intake frequency | 1558 | Alcohol intake frequency |
| Smoking | 20116 | Smoking status |
