# Supplementary material for: Associations Between Chronotype, Genetic Susceptibility and Risk of Colorectal Cancer in UK Biobank
Source: J Epidemiol Glob Health. 2025 Apr 10;15(1):57. doi: 10.1007/s44197-025-00399-6 (PMC11985712; doi:10.1007/s44197-025-00399-6)
Supplement: Supplementary file 3 — Supplementary file3 (DOCX 14 KB) [file 44197_2025_399_MOESM3_ESM.docx]

| Table S3. Data fields and International Classification of Disease Codes 10 used for identification of CRC and date of CRC diagnosis in the UK Biobank cohort | |
| --- | --- |
| Definition | Code |
| Colorectal cancer | C18 Malignant neoplasm of colon |
|  | C18.0 Caecum |
|  | C18.1 Appendix |
|  | C18.2 Ascending colon |
|  | C18.3 Hepatic flexure |
|  | C18.4 Transverse colon |
|  | C18.5 Splenic flexure |
|  | C18.6 Descending colon |
|  | C18.7 Sigmoid colon |
|  | C18.9 Colon, unspecified |
|  | C19 Malignant neoplasm of rectosigmoid junction |
|  | C20 Malignant neoplasm of rectum |
|  | C21 Malignant neoplasm of anus and anal canal |
|  | C21.0 Anus, unspecified |
|  | C21.1 Anal canal |
|  | C21.2 Cloacogenic zone |
|  | C21.8 Overlapping lesion of rectum, anus and anal canal |
| Date of cancer diagnosis (national cancer registry) | 40005 |
| Date of first in-patient diagnosis - ICD10 (hospital inpatient) | 41280 |
| Prevalent cancer within the UK Biobank cohort was identified through national cancer registries (data fields: 40006) and hospital inpatient records (data fields: 41270). | |
| Abbreviations: CRC, colorectal cancer; ICD, International Classification of Diseases. | |
