# Supplementary material for: Associations Between Chronotype, Genetic Susceptibility and Risk of Colorectal Cancer in UK Biobank
Source: J Epidemiol Glob Health. 2025 Apr 10;15(1):57. doi: 10.1007/s44197-025-00399-6 (PMC11985712; doi:10.1007/s44197-025-00399-6)
Supplement: Supplementary file 4 — Supplementary file4 (DOCX 14 KB) [file 44197_2025_399_MOESM4_ESM.docx]

| Table S4. Crosstab between Chronotype and Polygenic risk score | | | |
| --- | --- | --- | --- |
|  | Polygenic risk score | | |
|  | Low | Immediate | High |
| Chronotype | n (%) | n (%) | n (%) |
| Early chronotype | 37838 (63.88) | 111872 (63.05) | 22205 (37.6) |
| Late chronotype | 21394 (36.12) | 65568 (36.95) | 36852 (62.4) |
