# Supplementary material for: Associations Between Chronotype, Genetic Susceptibility and Risk of Colorectal Cancer in UK Biobank
Source: J Epidemiol Glob Health. 2025 Apr 10;15(1):57. doi: 10.1007/s44197-025-00399-6 (PMC11985712; doi:10.1007/s44197-025-00399-6)
Supplement: Supplementary file 5 — Supplementary file5 (PDF 102 KB) [file 44197_2025_399_MOESM5_ESM.pdf]

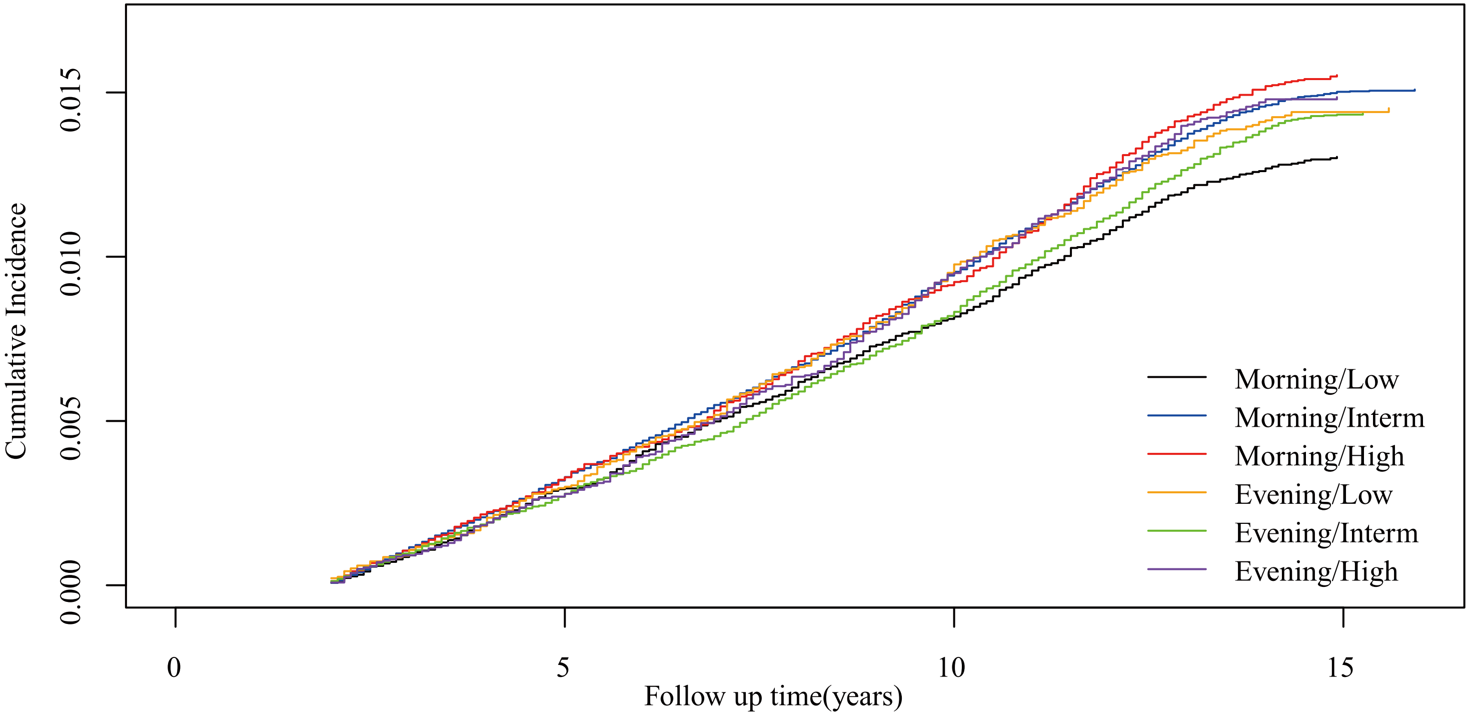

Figure S1. Cumulative Incidence of Colorectal Cancer Across Different Chronotype and PRS Groups
